# Enhancing prosthetic hand control: A synergistic multi-channel electroencephalogram

**DOI:** 10.1017/wtc.2024.13

**Published:** 2024-11-28

**Authors:** Pooya Chanu Maibam, Dingyi Pei, Parthan Olikkal, Ramana Kumar Vinjamuri, Nayan M. Kakoty

**Affiliations:** 1 Embedded Systems and Robotics Lab, Tezpur University, Tezpur, Assam, India; 2 Vinjamuri Lab, University of Maryland, Baltimore County, Baltimore, MD, USA

**Keywords:** independent component analysis, brain–computer interface, time-domain, support vector machine, prosthetic hand

## Abstract

Electromyogram (EMG) has been a fundamental approach for prosthetic hand control. However it is limited by the functionality of residual muscles and muscle fatigue. Currently, exploring temporal shifts in brain networks and accurately classifying noninvasive electroencephalogram (EEG) for prosthetic hand control remains challenging. In this manuscript, it is hypothesized that the coordinated and synchronized temporal patterns within the brain network, termed as brain synergy, contain valuable information to decode hand movements. 32-channel EEGs were acquired from 10 healthy participants during hand grasp and open. Synergistic spatial distribution pattern and power spectra of brain activity were investigated using independent component analysis of EEG. Out of 32 EEG channels, 15 channels spanning the frontal, central and parietal regions were strategically selected based on the synergy of spatial distribution pattern and power spectrum of independent components. Time-domain and synergistic features were extracted from the selected 15 EEG channels. These features were employed to train a Bayesian optimizer-based support vector machine (SVM). The optimized SVM classifier could achieve an average testing accuracy of 94.39 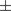 .84% using synergistic features. The paired *t*-test showed that synergistic features yielded significantly higher area under curve values (*p* < .05) compared to time-domain features in classifying hand movements. The output of the classifier was employed for the control of the prosthetic hand. This synergistic approach for analyzing temporal activities in motor control and control of prosthetic hands have potential contributions to future research. It addresses the limitations of EMG-based approaches and emphasizes the effectiveness of synergy-based control for prostheses.

## 1. Background and summary

The field of prosthetic hand control has witnessed remarkable advancements in recent years, aiming to enhance the functionality and usability of prosthetic hands by individuals with hand amputation. In research on motor control, electromyogram (EMG) has been a cornerstone enabling users to manipulate prosthetic hands through the detection of residual muscle activity (Campbell et al., 2020). However, EMG-controlled prostheses heavily rely on the availability and functionality of residual muscles. For individuals with amputations or muscle impairments, this reliance poses challenges in achieving consistent and reliable control (Fleming et al., 2021). Additionally, the overall versatility of prosthetic hand movements in EMG-controlled systems is constrained by the number of accessible muscle and their associated contractions. The complexity of muscle-based interfaces hinders the naturalness and speed of interaction. The users of EMG-controlled prostheses often face a learning curve associated with specific muscle contractions for precise control (Kumar et al., 2019).

With the advancement of neuroscientific understanding and technology, brain–computer interface (BCI) system with an electroencephalogram (EEG) serves as a means of enhancing the control precision and usability of prosthetic devices (Abiri et al., 2019). This will establish a direct channel for communication and control between the human brain and external devices, eliminating the need for reliance on peripheral nerves and muscles. Myokinetic interfaces were introduced for controlling prosthetic hands that rely on detecting muscle movements using surgically implanted sensors in or near the muscles (Gherardini et al., 2023). This technique presents an alternative to using scalp EEG for prosthetic hand control. However, this interface involves surgically implanting sensors to detect muscle activity posing several challenges. These challenges include the invasiveness of the surgery, risks of infection, rejection, and long-term biocompatibility issues (Salminger et al., 2019). Additionally, the implanted sensors can cause discomfort for users and require periodic maintenance or replacement resulting in higher costs. However, EEG provides comprehensive motor and cognitive activity insights without the associated risks and complexities of myokinetic interfaces. Bypassing the need for intact muscles, EEG offers an alternative for individuals with nonfunctional or absent muscles, broadening the scope of prosthesis accessibility. EEG allows for the decoding of more intricate neural patterns, potentially offering a greater range of distinguishable hand movements (Hazrati & Erfanian, 2010). Furthermore, EEG-based control strategies are more anthropomorphic enabling the interaction between the user and the prosthetic hand intuitively. Previous research has highlighted distinctions in EEG patterns associated with various hand movements such as opening, closing, supination, and pronation (Zou et al., 2023). Additionally, differences have been observed among different types of grasps, including pincer, power, and intermediate grasps (Su et al., 2023). However, these control strategies using EEG have limitations and challenges in detecting complex hand movements in higher dimensions (Zou et al., 2023; Su et al., 2023). EEG acquisition and processing require further engineering to integrate smoothly with prosthetic hands. To address these challenges, synergies are being explored that mimic the intelligent approach employed by the human brain for motor control (Santello et al., 2013). Studies have demonstrated the effective identification of synergies in hand movement (Antuvan et al., 2016).

Moreover, the complex functional structure of cortical motor areas and the reasons for their numerous interaction during voluntary movement remain not fully elucidated. Neurons within these regions possess distinctive characteristics and engage in diverse stages of movement, from planning to execution through functional integration (Andres & Gerloff, 1999; Tononi et al., 1998). Hand synergy correlation in kinematic movement (Pei et al., 2020), hand movement classification (Erdoĝan et al., 2019), effects of beta rebound, and alpha-coherence during execution of hand movements (Wang et al., 2022; Formaggio et al., 2015) have been reported involving EEG channels spanning over central, frontal, occipital, and parietal regions. Studying the synchronization of temporal patterns of the parallel pathways connecting frontal, central, parietal, and occipital regions of the brain provides an understanding of their dynamic roles in voluntary hand movement. Previous studies have shown that the analysis of muscle synergy is employed to investigate the coordination of signals associated with muscle activity (Santello et al., 2016; Li et al., 2021). The muscle synergy theory is grounded in the concept that the highly redundant musculoskeletal system requires a mechanism to streamline the degrees of freedom in motor control. This mechanism enables the execution of movements. The relevance of muscle synergy analysis becomes particularly evident in studies focusing on motor impairment. Currently, there is no method that ensures consistent categorization of distinct hand movements based on synergy in the brain using noninvasive EEG.

In this study, it is hypothesised that coordinating temporal patterns within the different regions of the brain, referred to as brain synergy, contain sufficient information for decoding various hand movements for prosthetic hand control. 32-channels EEG were recorded from 10 healthy participants during hand grasp and open tasks. Movement-related synergistic brain activity was analyzed using coherence of spatial power distribution pattern and power spectral density (PSD) of independent components of EEG. From the 32 channels, 15 channels located in the frontal, central, and parietal regions were selected using independent component analysis (ICA) based spatial power distribution and power spectral analysis to accurately decode the neural activity associated with hand movements. Time-domain and synergistic features (coherence of spatial power distribution and power spectral) were extracted from the selected 15 channels. These features were used to train a support vector machine (SVM) classifier. The SVM classifier was optimized using a Bayesian optimizer. The optimized SVM classifier achieved an average testing accuracy of 94.39 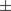 .84% across 10 participants using synergistic features. As a proof of concept, the output of the classifier was utilized to control a prosthetic hand. This synergistic method for analyzing temporal neural activities in motor control and controlling prosthetic devices offers promising contributions to future research in synergy-based prosthetic control.

## 2. Materials and methods

### 2.1. Human participants

Studies have shown that motor intentions and signals can still be detected after limb loss (Reilly et al., 2006). Individuals with hand amputation sometimes experience sensations where they perceive the presence of the lost limb (Wijk & Carlsson, 2015). These sensations are generated by the same motor cortical areas that were active before limb loss and thereby indicate the capability to generate motor signals. Furthermore, studies have shown that the motor intentions and signals remain the same for both healthy and individuals with limb amputations (Bruurmijn et al., 2017; Chen et al., 2013). Following these, our study involved 10 right-handed healthy participants, consisting of four males and six females, with an average age of 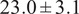 years. All procedures were conducted in compliance with relevant standards and regulations. The experimental protocol received approval from the Institutional Review Board at the Stevens Institute of Technology under Protocol No. 2015-022(20-AR6). Prior to the experiment, all participants provided informed written consent, demonstrating their understanding and agreement to participate in the study.

### 2.2. Task performance and data acquisition

**Figure 1.**
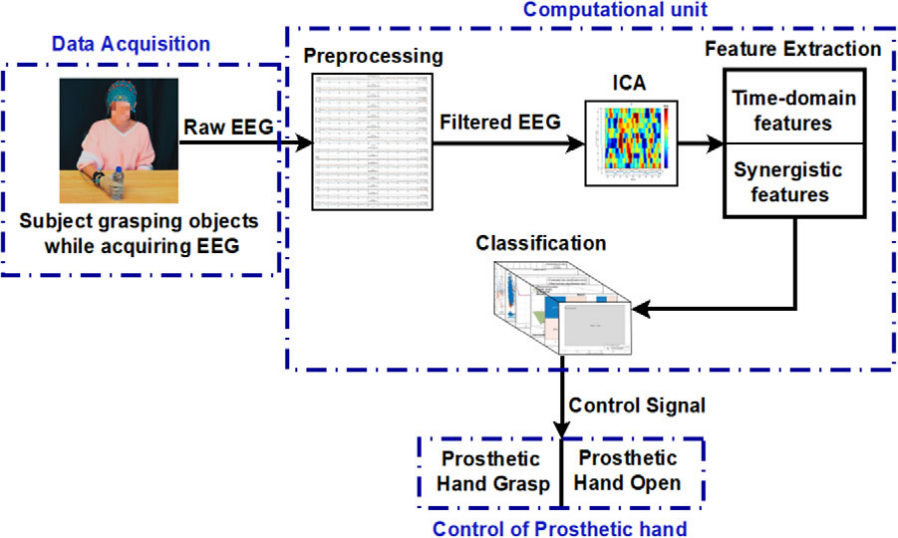
The schematic of the proposed method consists of data acquisition, computational unit, and interfacing with the prosthetic hand for the control of hand open and close tasks.

In the experimental setup for data acquisition, an EEG cap (g.GAMMA cap from g.tec, Schieldberg, Austria), an EEG electrode set known as g.Ladybird, and BCI2000 EEG acquisition system (Schalk et al., 2004) were employed. Additionally, a computational unit and a cylindrical water bottle were integral parts of the setup. The schematic representation of the experimental methodology employed in this study is illustrated in Figure 1. To evaluate the SVM classifier used for the classification of hand movement, a prosthetic hand (Kakoty et al., 2022) controlled using the classification results was employed. The EEG was acquired with 10/20 configuration 32-active high-density electrodes covering central, frontal, parietal, and occipital areas and additional intermediate positions (In1, In2, In3, In4, In5, In6, In7, and In8). The reference electrode was positioned on the earlobe, either at the left (A1) or right (A2), with the ground electrode on the nasion (NZ).

**Figure 2.**
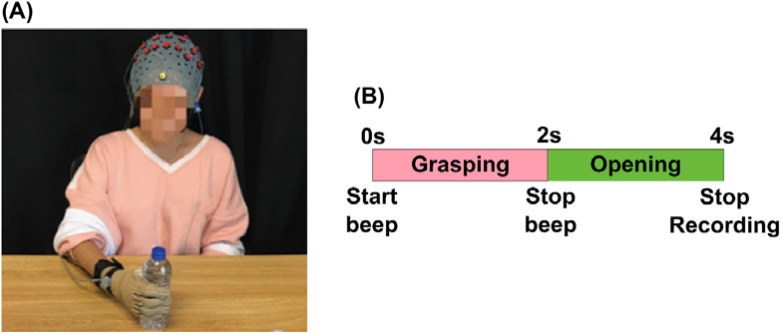
Experimental setup and timeline. (a) The illustration of a participant grasping an object during EEG acquisition (image has been adapted from (Pei et al., 2019)). (b) Experimental timeline for participants to perform the grasping and opening tasks.

Following the EEG cap setup, participants were seated in a relaxed position placing their dominant hand palm-down on the table. A water bottle was placed 40 cm away from the body midline of the participant to grasp comfortably as presented in Figure 2(a). The experimental timeline, illustrated in Figure 2(b), guided the participants through the task. Upon hearing an initiation signal from the computational unit, the participants were directed to grasp the cylindrical water bottle and release it when a subsequent signal was heard. The recording duration was set at 4 s, and each participant performed the experiment 30 times. Subjects were advised to minimize blinking and swallowing to mitigate potential artifacts.

### 2.3. Data and statistical analysis

#### 2.3.1. Data preprocessing

**Figure 3.**
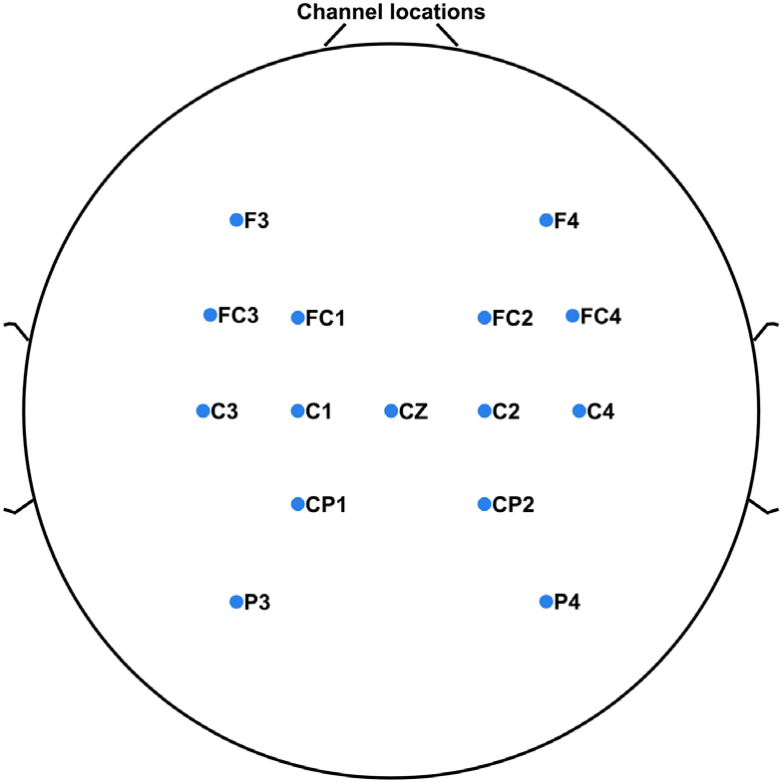
The spatial distribution of the selected 15 EEG channels across the scalp for this study. The specific locations of these channels are critical for the accurate analysis of EEG, ensuring a representative sampling of neural activity from frontal, central, parietal, and occipital areas.

The EEG was recorded continuously with electrode impedance maintained below 10 kOhms and 256 Hz sampling rate, capturing the temporal dynamics of brain activity at a high resolution. To enhance data quality, trials containing artifacts due to eye blinking or swallowing were rejected. Furthermore, the raw EEG data underwent a fourth order Butterworth bandpass filter from a frequency range between .53 and 60 Hz. The utilization of a Butterworth filter (Subasi & Gursoy, 2010), helped to achieve a smooth and effective filtering of the EEG signals, contributing to the precision of subsequent analyses. This specific frequency range was chosen to focus on the relevant neural activity while attenuating unwanted noise and potential artifacts. ICA was applied to the 32 channels of filtered EEG data to separate them into independent components (ICs) in EEGLAB v 2022.1 (Lee et al., 1999; Delorme & Makeig, 2004). The spatial power distribution pattern associated with each ICs are examined. Furthermore, channels exhibiting task-relevant patterns of PSD across the ICs are also identified. Based on the coherence of both the spatial power distribution patterns and the PSD of ICs, 15 channels corresponding to these ICs are selected. The acquired 32-channel EEG were narrowed down to a subset of 15 channels for the classification of hand grasp and open tasks. These selected 15 channels span the frontal, central, and parietal regions of the human brain. The spatial distribution of the selected EEG channels across the scalp is depicted in Figure 3. This strategic placement of electrodes ensures a representative sampling of neural activity, effectively covering key regions that are directly influenced during the initiation and execution of hand movement indicating brain synergy (Erdoĝan et al., 2019; Teplan, 2002; Wang et al., 2022; Pei et al., 2020).

#### 2.3.2. Feature extraction in the time domain

Eleven time-domain features were extracted from the filtered EEG using a window size of .5 s with an overlap of .3 s (Meier et al., 2008). The extracted time-domain features are minimum value (*min*), maximum value (*max*), median amplitude (*med*), mean amplitude ( 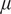 ), standard deviation ( 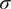 ), variance (*var*), waveform length (*WL*), mean absolute value (*MAV*), root mean square ( 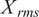 ), skewness (*skew*), and kurtosis (*kur*). These features encompass crucial aspects of EEG signals, including amplitude, regularity, and synchronization (Emigdio et al., 2016; Olcay & Karaçalı, 2019). A 9,000 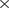 11 feature matrix was created to classify hand movements. Each row in the matrix corresponds to 10 individuals conducting hand open and grasp operations. Each subject performed the task 30 times, resulting in 600 trials. The matrix includes data from 15 EEG channels, resulting in 9,000 data points. Each column represents the 11 extracted features across 15 selected EEG channels. The details of the extracted time domain features were reported in (Pooya Chanu et al., 2023).

#### 2.3.3. Computation of power spectral density

EEG signals collected during grasp and open tasks were segmented into specific time windows. These time windows were .5–1 s, 1.5–2 s, 2.5–3 s, and 3.5–4 s. ICA was employed on the segmented data to decompose the EEG data into ICs where each component represented a distinct neural source. After extracting ICs using ICA, the PSD of each IC was calculated by applying the Fourier transform using Welch’s method (Zhao & He, 2013). PSD values were computed for each frequency band, including Broadband (4–100 Hz), theta (3–8 Hz), alpha (8–12 Hz), beta (13–35 Hz), and gamma (36–100 Hz). The frequency range for each band was determined based on previous literature cited (Ali et al., 2022; Van Albada & Robinson, 2013). The coherence of spatial power distribution pattern and PSD during hand grasp and opening were estimated from the ICs. The analysis resulted in 54,000 synergistic features per window, considering all frequency bands across 15 EEG channels.

### 2.4. Classification

The recorded EEG data results in a high-dimensional feature space. The versatility of kernel selection in SVM allows for nonlinear mapping of input features to higher dimensional spaces, making it suitable for EEG data. The SVM operates by transforming the training dataset through mapping to a nonlinear vector space of high dimensions, accommodating the complexity of EEG data (Lotte et al., 2007). Additionally, SVM is known for its robustness to overfitting which is a crucial consideration in EEG analysis (Hosseini et al., 2023; Thanigaivelu et al., 2023; Martínez-Ramón et al., 2006). Based on these, the SVM classifier was chosen as the model for classification in this study.

For the classification training phase, two sets of features were utilized: time-domain features and synergistic features. Initially, the SVM classifier was trained using a combined matrix of time-domain features, which encompassed class labels for hand grasp and open states. The trials of each participant were divided into 24 folds to ensure robust training and evaluation. Subsequently, the synergistic features were employed for classification training using the SVM classifier. This involved a similar methodology to the time-domain feature training incorporating synergistic information.

To further enhance the performance of the SVM classifier, hyperparameter optimization was performed. Given the significant impact of internal parameters on SVM effectiveness, Bayesian optimizer (Luo et al., 2020) was employed for optimization of SVM. The objective function for training the model was defined as the mean square error. The Bayesian optimizer iteratively adjusted the hyperparameters within specified search ranges to minimize error in cross-validation. This approach ensured that the SVM classifier was fine-tuned for optimal performance in classifying EEG data.

**Figure 4.**
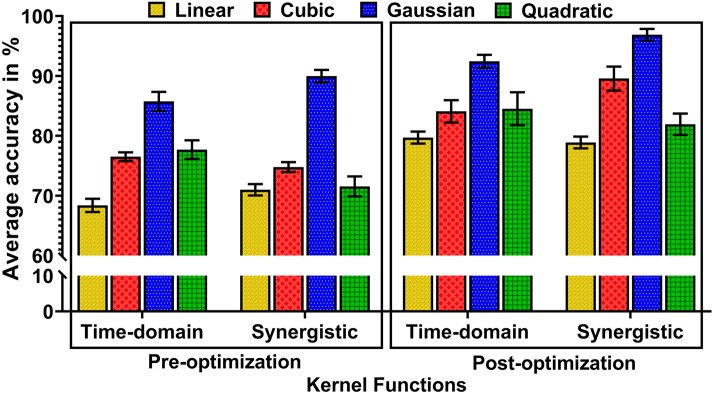
Comparative plot of classification accuracy using different kernel functions pre-optimization and post-optimization across 10 participants.

Figure 4 depicts the average training classification accuracy of linear, cubic, Gaussian, and quadratic kernel functions, both before and after optimization with standard deviation. It utilizes time-domain and synergistic features from 10 participants. Averaging the training set, the minimized classification error was computed considering the input-to-output potential mappings. The adaptation of optimal values for internal parameters underscores the significance of hyper-parameters in minimizing prediction error. The highest accuracy of classification during training was achieved using gaussian kernel with both time-domain and synergistic features.

## 3. Results and discussion

### 3.1. SVM optimization

**Figure 5.**
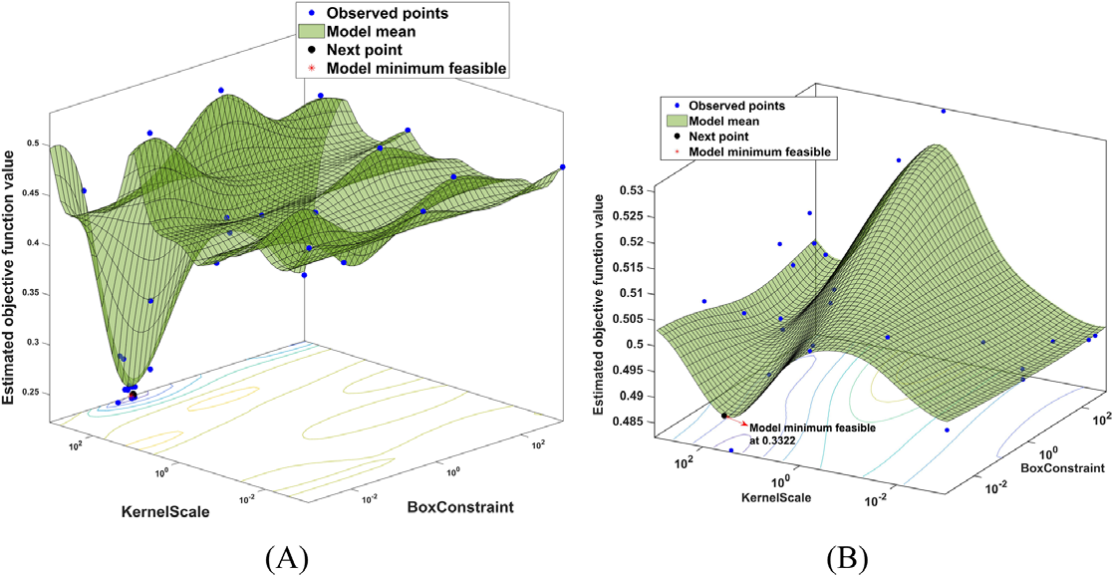
The SVM hyper-parameters optimization using a Bayesian optimization approach. (A) The optimized hyper-parameters were obtained for time domain feature-based classification. (B) The optimized hyper-parameters were obtained for synergistic feature-based classification.

Figure 5(a and b) depicts the SVM hyperparameter optimization using Bayesian optimization for time domain synergistic features-based classification. The green surface illustrates the performance at different hyperparameter points, while the sampled parameter space is in blue points. The estimated objective function value corresponds to the misclassification rate. The hyperparameter values in the optimized SVM that resulted in the lowest misclassification error during the training phase were identified. The optimized values of the SVM classifier hyperparameter were achieved with Gaussian kernel, kernel scale of 341.21 and box constrain level of 5.93. The optimized values for synergistic features with Gaussian kernel are a kernel scale of .87 and a box constraint level of 271.80. The optimized misclassification rate with time-domain and synergistic features are 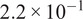 and 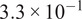 respectively during training of the optimized SVM. This optimized configuration enhances the performance of the SVM classifier, considering both time-domain and synergistic features.

### 3.2. Spatial distribution of power

**Figure 6.**
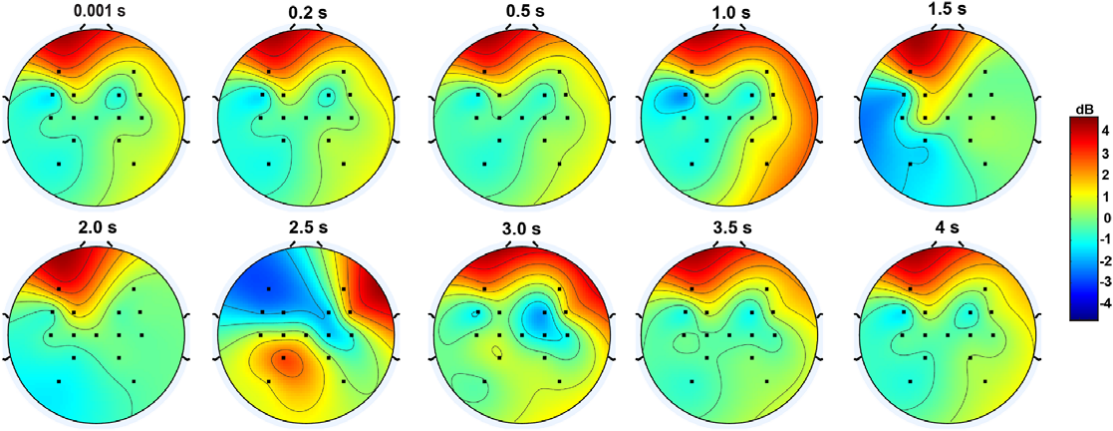
Spatial distribution of power (in decibels (dB) with time (in s) during hand grasp and open states across 10 participants.

Studies have shown that investigation of the spatial distribution of power in different regions of the brain demonstrates the specific neural networks engaged during hand movements (Andres & Gerloff, 1999; Tononi et al., 1998). The identification of task-specific activation patterns during hand grasp and open tasks contributes to characterizing distinct motor control processes. The spatial distribution of power, expressed in decibels (dB), over time (in s) during hand grasp and open across 10 participants is depicted in Figure 6. A dynamic changes in the power levels and their spatial patterns was observed during hand grasp and opening. However, no distinct difference was observed during the initiation of hand grasp and execution of hand opening. During the initiation of hand opening at 2.5 s, the increase in power in F4, FC4, FC2, CP1, and P3 shows the formulation of motor commands necessary to perform the hand open task and continuous sensory feedback in refining the movement.

### 3.3. Temporal neural coordination

**Figure 7.**
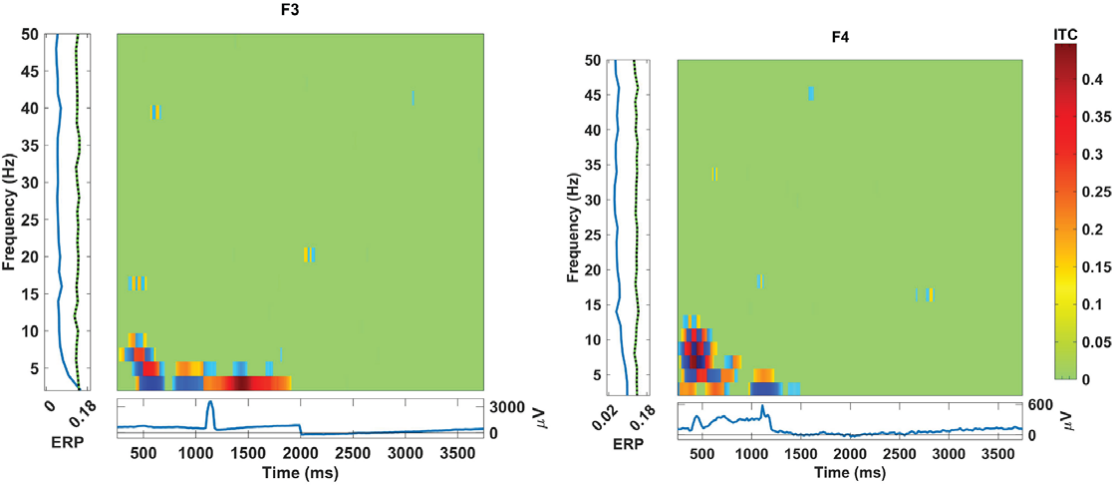
ITC during hand grasping and opening in the EEG channel located at the frontal region with the significance values of .01.

**Figure 8.**
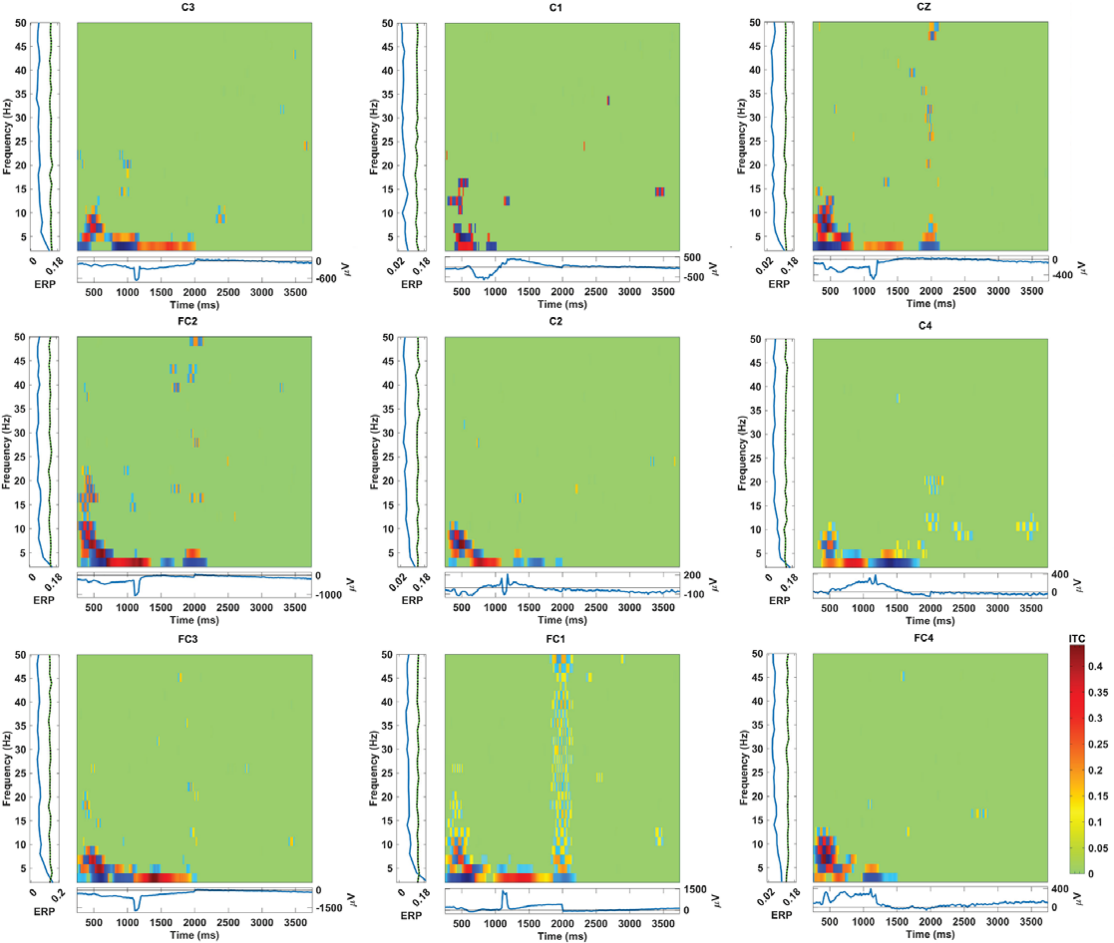
ITC during hand grasping and opening in the EEG channel located at the central region with the significance values of .01.

**Figure 9.**
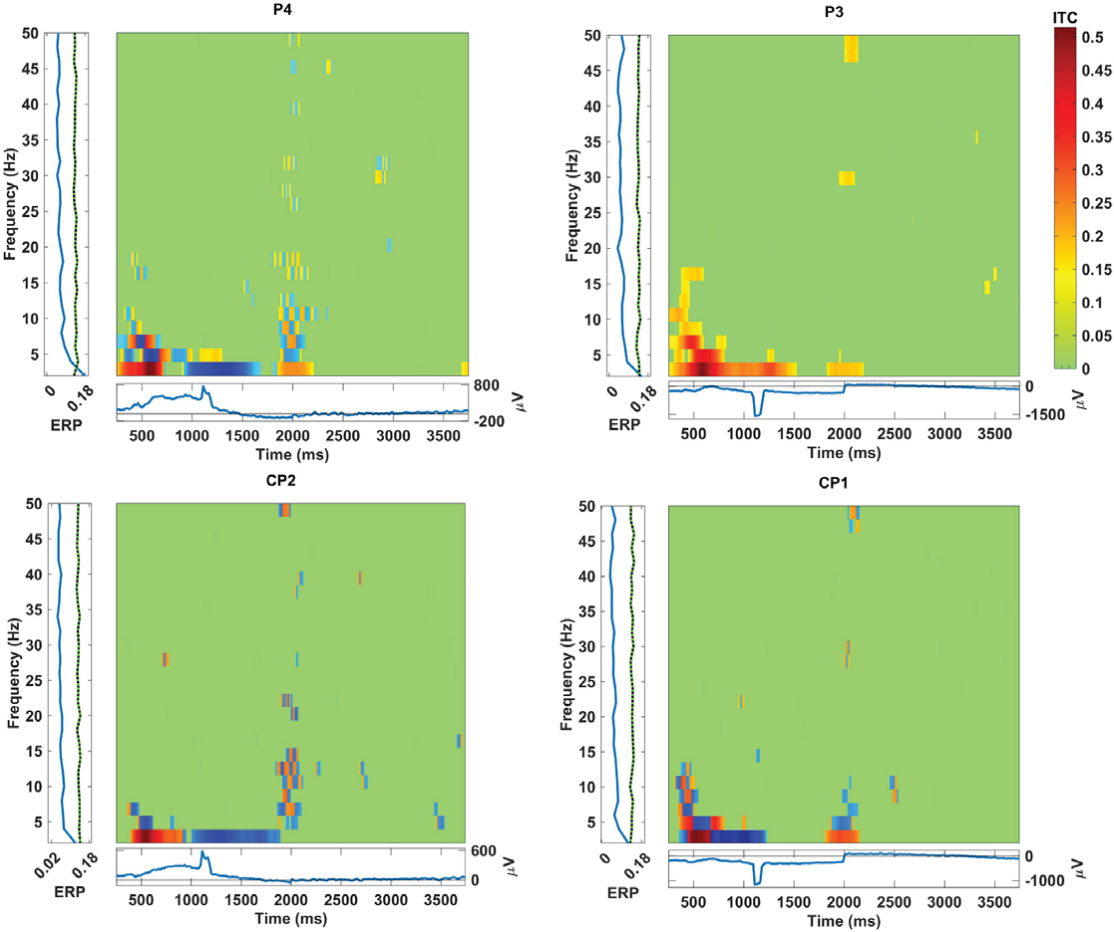
ITC during hand grasping and opening in the EEG channel located at the parietal region with the significance values of .01.

The graphs depicting inter-trial coherence (ITC) with a significance level of .01 during hand grasp and open for the EEG channels located in the frontal, central, and parietal regions are shown in Figures 7–9. The *x*-axis represents time in ms, while the *y*-axis denotes the coherence values, reflecting the degree of synchronization among the brain regions. The scale on the right of the ITC plots ranging from green to red indicates the magnitude of coherence and the blue squares present in the graph indicate no significant coherence. Furthermore, Figures 7–9 show the event-related potentials (ERP) and frequency power changes over time during hand grasp and open. The ITC analysis illustrates the temporal dynamics of neural synchronization during the hand grasp and open task providing insights into the coordinated activity across different brain regions. This significance level indicates a high level of confidence in the coherence values in which the observed synchronization patterns are unlikely due to random chance. These results highlight the temporal dynamics of synergy between different EEG channels during the motor tasks for visual comparison of coherence patterns. In Figure 7, the ITC values indicate consistent engagement in synchronized neural activity in the frontal region during hand grasp. Additionally, distinct peaks in the ERP waveforms show active neural processing in the frontal region for planning and decision-making involved in initiating and executing hand movements. In Figure 8, the ITC values for the central region show strong synchronization and neural activation during hand movements. The high ITC values observed in Figure 8 reflect significant engagement of the motor cortex, with the ERP waveforms indicating the direct involvement motor cortex in controlling and executing grasp tasks in a time-locked manner. In Figure 9, the ITC values for the parietal region exhibit significant synchronization patterns with high coherence. The ERP alterations in this region indicate its role in guiding motor responses during hand grasp and open tasks with sensory feedback. These findings collectively illustrate the coordinated neural activity known as synergy across different brain regions during hand movements with ITC and ERP analyses.

**Figure 10.**
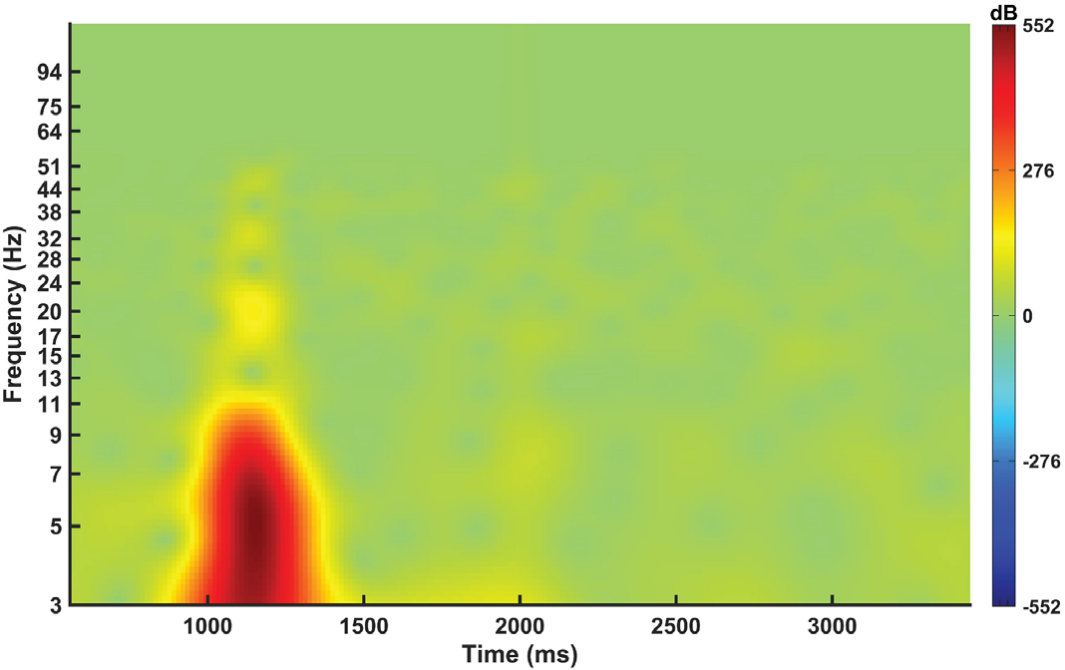
Spatial distribution of power (in decibels (dB) with time (in s)) during hand grasp and open across 10 participants.

The frequency versus time plot for EEG during hand grasp and open tasks is presented in Figure 10. This result shows the temporal variations in neural activity, highlighting a surge in power within 3–8 Hz frequency range during the hand grasping phase. This elevation in power in the theta frequency band signifies intensified neural engagement associated with motor planning, execution, and sensorimotor integration of voluntary movements. The observed increase in power in theta range (3–8 Hz) reflects the complex neural processes involved in hand grasping. The frequency versus time plot not only captures variations in power but also provides insights into the temporal dynamics and functional connectivity patterns contributing to the successful execution of the hand grasp task. However, during the hand-opening phase, distinct neural activation patterns are not observed with no power surge. The neural activation diminishes after 2 s of hand grasp. The distinct surge in power within the theta frequency range (3–8 Hz) during hand grasp and no power surge during hand opening shows the more intensive neural engagement due to the need for precise motor control and sensorimotor feedback integration. This neural activity can be a critical feature for differentiating hand grasp and open task classification. These results underscore the importance of considering both the temporal dynamics and frequency-specific power changes in neural activity for effective synergistic classification of hand movements. Studies reported that during hand grasp, the complex network of cortical areas is involved in planning and execution leading to a significant rise in theta activity (Goldenkoff et al., 2023; Iturrate et al., 2018). However, during hand opening, the less complex motor planning and execution were involved compared to hand grasp (Goldenkoff et al., 2023). Therefore, there may not be a significant rise in theta activity as the demands on the cortical grasping network and associated neural populations are less.

### 3.4. Evaluation of classifier performance

**Figure 11.**
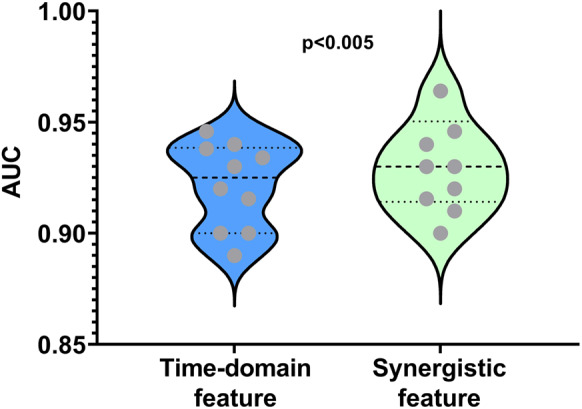
AUC values indicate the performance of 10 participants using time-domain and synergistic features with *p* < .005. Grey dots = individual performance AUC values, thick dotted line = group mean.

The analysis of the area under the curve (AUC) for the classifier trained using the time domain and synergistic features presented in Figure 11 provides valuable insights into the SVM classifier in the context of distinguishing hand movement classes. The AUC value for synergistic features is .945 which is higher than the AUC for time-domain features which .926. The resulted values of AUC value indicates that the classifier is optimal for classifying the hand movement dataset as reported in (Metz, 1978). This distinction suggests that synergistic features play a more effective role in enhancing the accuracy of a classifier to discern subtle variations in hand movement patterns.

Z-score normalization was applied to both the synergistic and time-domain features to standardize each feature. A paired *t*-test was performed to compare the performance of classifiers trained with the normalized time-domain features versus synergistic features. This test evaluates the significance of the difference in AUC values between the two classifiers. The results revealed a *p*-value < .05, indicating that the difference in AUC values is statistically significant. This finding shows that synergistic features offer a more accurate and reliable basis for classifying hand movement patterns compared to time-domain features. The reliance of the SVM classifier on synergistic features highlights the significance of frequency-specific information in capturing the neural signals associated with distinct motor activities. This finding has practical implications for the design and optimization of classifiers in applications for synergy-based prosthetic control.

**Figure 12.**
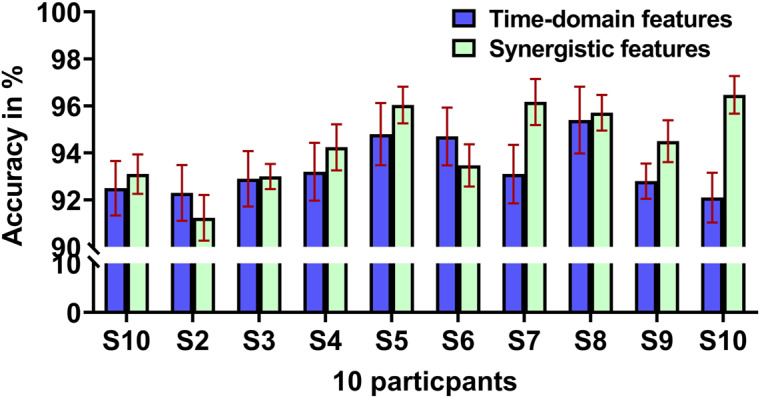
Classification accuracy of 10 participants with time domain features and synergistic features.

For the classification of hand grasp and open using the SVM classifier, 80% of the dataset was randomly selected for training, while the remaining 20% was used for testing. This division of the dataset ensures a comprehensive evaluation of the performance of the classifier. This leveraged a significant portion of the data for training while reserving a sufficient sample for testing to validate the accuracy of the classifier. The classification accuracy using time-domain and synergistic features of 10 participants is presented in Figure 12. During the training, the classifier exhibits an accuracy of 96.8 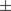 .98% with time-domain features. During testing of the classifier, an average accuracy of 93.4 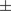 1.16% was obtained across 10 participants. The lowest accuracy of classification was 92.1% in the S10 and the highest accuracy of 95.4% was observed in S8.

In the second set of classifications with synergistic features, the classifier maintained a high accuracy of 97.1 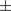 .9134% during the training and achieved an accuracy of 94.39 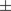 .8434% during testing. The classification accuracy varied among subjects, with the highest accuracy recorded for S10 at 96.47% and the lowest accuracy observed in S2 at 91.24%. These findings underscore the robustness of the classifier across different sets of data, indicating its adaptability and effectiveness in consistently distinguishing between hand grasp and open task.

### 3.5. Proof-of-concept

The optimized SVM classifier used for the classification of hand grasp and open was deployed to control prosthetic hand grasping operations. A seamless interface was established between the output of the SVM classifier and a prosthetic hand for the visualisation of classification performance. The output of the SVM classifier framed in transistor-transistor logic was directed to an 8-bit microcontroller. The controller translates the output of the SVM classifier into the corresponding actuating signal. The actuating signal was transmitted to the actuators on the prosthetic hand through a current buffer circuit. The control circuit was powered by a standalone 9-volt lithium polymer battery.

**Figure 13.**
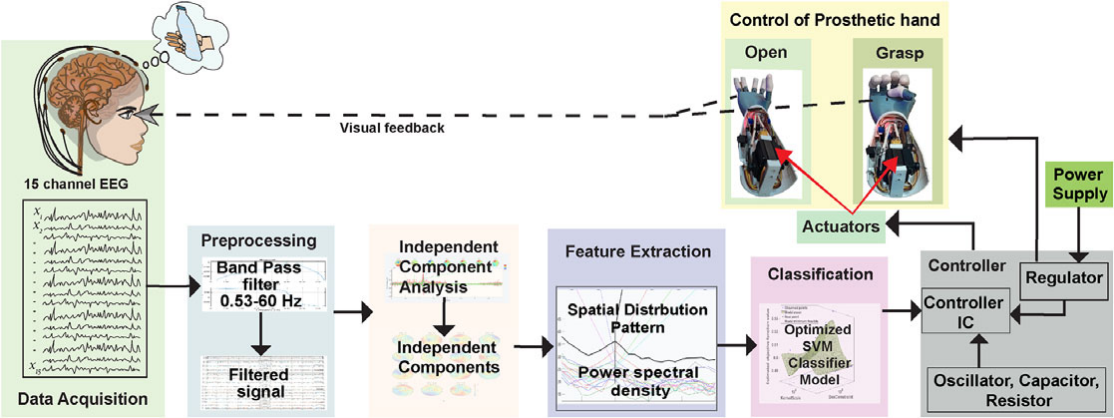
Prosthetic hand executing grasping and opening task with the optimized SVM classifier using synergistic features.

This controlled operation effectively demonstrated accurate classification performance by the optimized SVM. The integration of selected EEG channels, time-domain features and synergistic features underscored the versatility and adaptability of the SVM classifier. Figure 13 visually depicts the grasp and open task of the prosthetic hand, showcasing the successful implementation of the optimized SVM classifier in real-time prosthetic hand control. This innovative proof of concept not only validates the efficacy of the synergistic features-based SVM classification but also highlights its potential for practical applications in neuroprosthetics, offering a promising avenue for enhancing the lives of individuals with limb loss. However, the integration of EEG with prosthetic hand control presents significant challenges during both data acquisition and control of prosthetic devices. Noise from muscle activity and environmental factors distort neural signals, necessitating the use of artifact removal techniques. Variability in classification accuracy is influenced by fluctuations in EEG signals and the user, suggests increasing the number of participants for improved classification outcomes. While the integration of SVM classifier outputs with prosthetic hand actuators has shown promise in translating EEG into hand movement commands, residual noise in EEG and classification variability remain as a limiting factor. To overcome these challenges, advanced signal processing techniques, algorithm optimization, and the development of compact EEG systems will be essential in fully realizing the potential of EEG-based prosthetic control in practical applications.

## 4. Conclusions

The study demonstrated the complex neural dynamics associated with hand grasp and open tasks using EEG-based analysis. The spatial distribution of power across different brain regions during these tasks observed dynamic changes in power levels and spatial patterns. Inter-trial coherence analysis further reveals consistent synchronization patterns in the coordination of neural activity. Examining ERP and frequency dynamics during hand grasp and open tasks demonstrated temporal variations in neural activity. Notably, the alteration in power within the theta frequency range during hand grasp indicates neural engagement associated with motor planning and execution. The temporal dynamics and functional connectivity patterns revealed through these analyses contribute to our understanding of the synergy between different EEG channels during motor tasks.

The classifier performance analysis underscores the robustness of an optimized SVM classifier in distinguishing hand movement classes. Both time-domain and synergistic features contribute to high accuracy during training and robust generalization during testing. The higher AUC for synergistic features highlights their effectiveness in enhancing classifier accuracy, emphasizing the significance of frequency-specific information in capturing neural signals for hand movement. The high accuracy during training and robust generalization during testing underscores the potential of this approach for real-time applications in neuroprosthetics. These findings contribute to advancing the field, offering a promising avenue for enhancing the quality of life for individuals with limb loss through brain synergy-based control of prosthesis.

## Data Availability

Not applicable.
